# Outcomes of Radiotherapy in Oligoprogressive Breast Cancer

**DOI:** 10.3390/jpm14080805

**Published:** 2024-07-29

**Authors:** Fabio Marazzi, Valeria Masiello, Armando Orlandi, Francesca Moschella, Silvia Chiesa, Alba Di Leone, Giovanna Garufi, Ciro Mazzarella, Alejandro M. Sanchez, Calogero Casa, Angela Bucaro, Flavia De Lauretis, Niccolo Borghesan, Luca Tagliaferri, Gianluca Franceschini, Emilio Bria, Riccardo Masetti, Alessandra Fabi, Cynthia Aristei, Giampaolo Tortora, Vincenzo Valentini, Maria A. Gambacorta

**Affiliations:** 1 Fondazione Policlinico Universitario “A. Gemelli” IRCCS, UOC di Radioterapia Oncologica, Dipartimento di Diagnostica per Immagini, Radioterapia Oncologica ed Ematologia, 00168 Rome, Italy; fabio.marazzi@policlinicogemelli.it (F.M.); valeria.masiello@policlinicogemelli.it (V.M.); silvia.chiesa@policlinicogemelli.it (S.C.); ciro.mazzarella@policlinicogemelli.it (C.M.); calogero.casa@fbf-isola.it (C.C.); luca.tagliaferri@policlinicogemelli.it (L.T.); vincenzo.valentini@policlinicogemelli.it (V.V.); mariaantonietta.gambacorta@policlinicogemelli.it (M.A.G.); 2 Fondazione Policlinico Universitario “A. Gemelli” IRCCS, UOC di Oncologia Medica, Dipartimento di Scienze Mediche e Chirurgiche, 00168 Rome, Italy; armando.orlandi@policlinicogemelli.it (A.O.); giovanna.garufi@guest.policlinicogemelli.it (G.G.); emilio.bria@policlinicogemelli.it (E.B.); giampaolo.tortora@policlinicogemelli.it (G.T.); 3 Fondazione Policlinico Universitario “A. Gemelli” IRCCS, UOC di Chirurgia Senologica, Dipartimento di Scienze della Salute della Donna e del Bambino e di Sanità Pubblica, 00168 Rome, Italy; francesca.moschella@policlinicogemelli.it (F.M.); alba.dileone@policlinicogemelli.it (A.D.L.); ange.bucaro@gmail.com (A.B.); flavia.delauretis@gmail.com (F.D.L.); niccolo.borghesan@gmail.com (N.B.); gianluca.franceschini@policlinicogemelli.it (G.F.); riccardo.masetti@policlinicogemelli.it (R.M.); 4 Università Cattolica del Sacro Cuore, 00168 Rome, Italy; 5 Fondazione Policlinico Universitario “A. Gemelli” IRCCS, UOSD di Medicina Personalizzata in Senologia, Dipartimento di Scienze della Salute della Donna e del Bambino e di Sanità Pubblica, Direzione Scientifica, 00168 Rome, Italy; alessandra.fabi@policlinicogemelli.it; 6 Radiation Oncology Section, University of Perugia and Perugia General Hospital, 06156 Perugia, Italy; cynthia.aristei@unipg.it

**Keywords:** oligoprogression, breast cancer, radiotherapy, target therapies

## Abstract

Introduction: Radiotherapy (RT) shows potential for improving local control in cases of oligoprogressive metastatic breast cancer (mBC). This retrospective analysis aims to evaluate the advantages of RT in such a clinical scenario. Methods: We conducted a retrospective analysis including patients with mBC who received radiation therapy (RT) for up to three sites of oligoprogression while continuing systemic therapy. The study took place between January 2014 and December 2021. Our endpoints were progression-free survival after radiotherapy (PFS-AR), the rate of discontinuation of systemic therapy (RDT) at three months post-RT, and overall survival (OS). We used Cox regression analysis to perform multivariate analysis for PFS-AR. Results: Fifty-nine patients met the inclusion criteria. The PFS-AR was 13 months (95% CI 8.5–18.8 months). At three months, the RDT was 3% (two patients). A significant difference in median PFS-AR was observed between patients in the first + second-line group and those in the subsequent line group (*p* = 0.03). In the multivariate analysis conducted for PFS-AR, the biologically effective dose (BED) with α/β = 4 > 100 Gy emerged as the sole significant variable (*p* = 0.0017). The median overall survival (OS) was 24.4 months (95% CI 17–24.4 months). Conclusions: This study is the first report on the outcomes of radiotherapy in a cohort of over 50 patients with oligoprogressive metastatic breast cancer (mBC). Our findings emphasize the significant relationship between PFS-AR, the number of ongoing lines of systemic therapy, and the BED of radiotherapy.

## 1. Introduction

In recent years, there have been significant advancements in the treatment of metastatic breast cancer (mBC), leading to improved survival rates. However, the prognosis for mBC remains critical [1,2]. About 25–28% of patients are initially diagnosed with metastatic breast cancer, while 20–30% experience metastatic recurrence [3]. Systemic therapy is the standard treatment for mBC, with the goal of improving survival and enhancing patients’ quality of life [4]. The introduction of new targeted drugs and improvements in diagnostic imaging has led to the increased recognition of oligometastatic and oligoprogressive disease in clinical practice, prompting a reconsideration of classification and treatment approaches [3,4,5].

In the traditional approach to managing disease progression, patients switch to a new drug in order to improve progression-free survival (PFS) and overall survival (OS). However, when all systemic therapies have been tried and are no longer effective, it becomes challenging to control the disease, and patients may need to rely on supportive care only. Treating oligometastatic disease with local therapies has been established as a way to extend overall survival (OS) with low toxicity in specific diseases [6,7]. Oligoprogression, which is characterized by limited progression in a few lesions while the widespread metastatic disease remains stable [4,5,6,7,8], has emerged as a potential candidate for local treatment to extend overall survival [9,10,11]. In non-small cell lung cancer (NSCLC) with specific gene mutations, radiotherapy targeting local progression sites has become a standard approach, recommended in guidelines [12].

The existing research on oligoprogressive metastatic disease has primarily focused on OS or PFS as a result of combining systemic therapy with locoregional radiotherapy, or solely on local control (LC) [13]. However, there is a lack of data on metastatic breast cancer (mBC) in the context of oligoprogressive disease. Additionally, previous studies on mBC did not provide insights into the effectiveness of locoregional treatment across different subtypes [13]. This retrospective analysis aims to identify and characterize patients with oligoprogressive breast cancer who received radiotherapy without changing their systemic treatment, assess the role of local radiation treatment in prolonging PFS, and establish hypotheses for future prospective studies in this patient group.

## 2. Materials and Methods

In this analysis, the mBC cohort treated at our institute from January 2014 to December 2021 was retrospectively analyzed in order to select patients with oligoprogression who underwent local radiotherapy without changing ongoing systemic therapy. The Ethical Committee of Fondazione Policlinico Gemelli IRCCS approved the data collection and analysis (Ethical Committee of Fondazione Policlinico Gemelli IRCCS is 0023426/20, approved on 21 May 2020).

### 2.1. Patients’ Selection

The literature defines oligoprogression as “a patient who can have any number of metastases, as long as only a solitary or a select few show progression, with the rest displaying either regression or stability while the patient continues to receive systemic antitumor therapy” [14].

After an initially successful therapy for plurimetastatic disease, the state of oligoprogression take place when disease progression occurs only in a minority of the affected sites [13].

Before the study began, a multidisciplinary board consisting of medical and radiation oncologists established inclusion criteria. These criteria included the inclusion of mBC patients exhibiting up to three sites of oligoprogression treated with radiotherapy, continuation of systemic therapy after local treatment, availability of clinical and instrumental follow-up data, and at least one instrumental reassessment within three months following the completion of radiation therapy. Oligoprogression in this analysis was identified radiologically in accordance with RECIST/PERCIST criteria [15], using CT, MRI, and PET/CT scans conducted during follow-up evaluations.

Inclusion criteria were:

- Age ≥ 18 years;
- Histologically proven breast cancer (all subtypes included);
- Metastatic lesions pathologically or radiologically diagnosed;
- Ongoing systemic therapy (endocrine therapy, chemotherapy, and/or targeted therapy);
- Detection of oligoprogression during re-evaluation examinations while on systemic therapy, with patients undergoing radiotherapy for oligoprogressive sites without modifying systemic therapy until at least the next instrumental examination or until clinical progression appeared;
- Availability of follow-up data.

Exclusion criterion:

- All the patients with the same characteristics of the tumor that at oligoprogression time did not undergo radiotherapy and/or change systemic therapy ongoing.

### 2.2. Data Collection and Analysis

The data were collected retrospectively and anonymously using the GENERATOR Breast Data Mart [16]. After selecting the patients, information about primary and metastatic cancer, systemic therapies, local radiotherapy, and survival follow-up were recorded.

The collected data included age at diagnosis, clinical stage at diagnosis, cancer subtype, date of metastasis appearance, number of previous chemotherapy treatments for metastatic disease, current systemic treatments, radiotherapy details such as volumes and doses, end date of radiotherapy, date of disease progression after radiotherapy, date of last follow-up, and date of death. The subtypes were classified according to the St. Gallen Consensus of 2013 [17].

The radiotherapy treatments used different dosages and frequencies, in order to see if a specific treatment schedule was linked to PFS-AR. The treatments were converted into BED values using α/β ratios of 4 and 10. Then treatments were divided into four groups based on whether they were above or below 75 Gy and 100 Gy. The α and β values show how sensitive the irradiated cells are to radiation, with higher values meaning greater sensitivity.

The chosen cut-offs were based on the macroscopic breast cancer response observed at 75 Gy and the overall ablative effects of treatments delivering 100 Gy or more in BED, as explained in the discussion section.

The study had two primary goals: to assess progression-free survival after radiotherapy (PFS-AR) and to determine the rate of discontinuation of systemic therapy (RDT) three months after radiotherapy due to disease progression. PFS-AR was measured from the end of radiotherapy to the documented instrumental progression that required a change in systemic therapies or led to death. The secondary goal was to evaluate overall survival (OS), calculated from the primary diagnosis of breast cancer to the last clinical assessment or death.

The characteristics of the sample were presented using descriptive statistics. Quantitative variables were described using measures such as minimum, maximum, range, average, and standard deviation, while qualitative variables were displayed in tables showing absolute frequency and percentages.

Due to the varying doses and volumes used in radiotherapy, treatments were retrospectively converted into biologically effective doses (BED). We used both α/β = 4, as a measure of the sensitivity of breast cancer to radiation [18], and α/β = 10, as a measure of the sensitivity of metastases to radiation [1] for the conversion. We set the cut-offs at 75 Gy and 100 Gy, respectively, to differentiate treatments that specifically targeted secondary lesions from breast cancer [1] and those with a general ablative intent [19].

For the exploratory nature of the study, it was not possible to establish a statistical design. However, to evaluate the impact of radiotherapy treatment, we hypothesized that a PFS-AR greater than six months and an RDT less than 30% would be clinically relevant.

The analysis of PFS-AR and OS was conducted using Kaplan–Meier curve analysis with the log-rank test to highlight differences. Median values were reported with 95% confidence interval (CI). Further analyses for PFS-AR and OS were carried out based on age groups (<40 years, 40–50 years, 51–70 years, >70 years), subtypes (luminal A-like, luminal B-like, HER2+, and triple-negative) [17], HER2+ driver status (compared to all other groups), ongoing systemic line (number of systemic lines and first line + second line versus third line or more), site of oligoprogression treated with radiotherapy (type of parenchyma treated, brain compared to other sites), and biologically effective dose (BED) cut-offs (75 Gy α/β = 4 and α/β = 10, 100 Gy α/β = 4 and α/β = 10). All statistical analyses were performed using the MedCalc statistical platform, and *p*-values less than 0.05 were considered significant.

## 3. Results

### 3.1. Patients’ Characteristics

**Figure 1:**
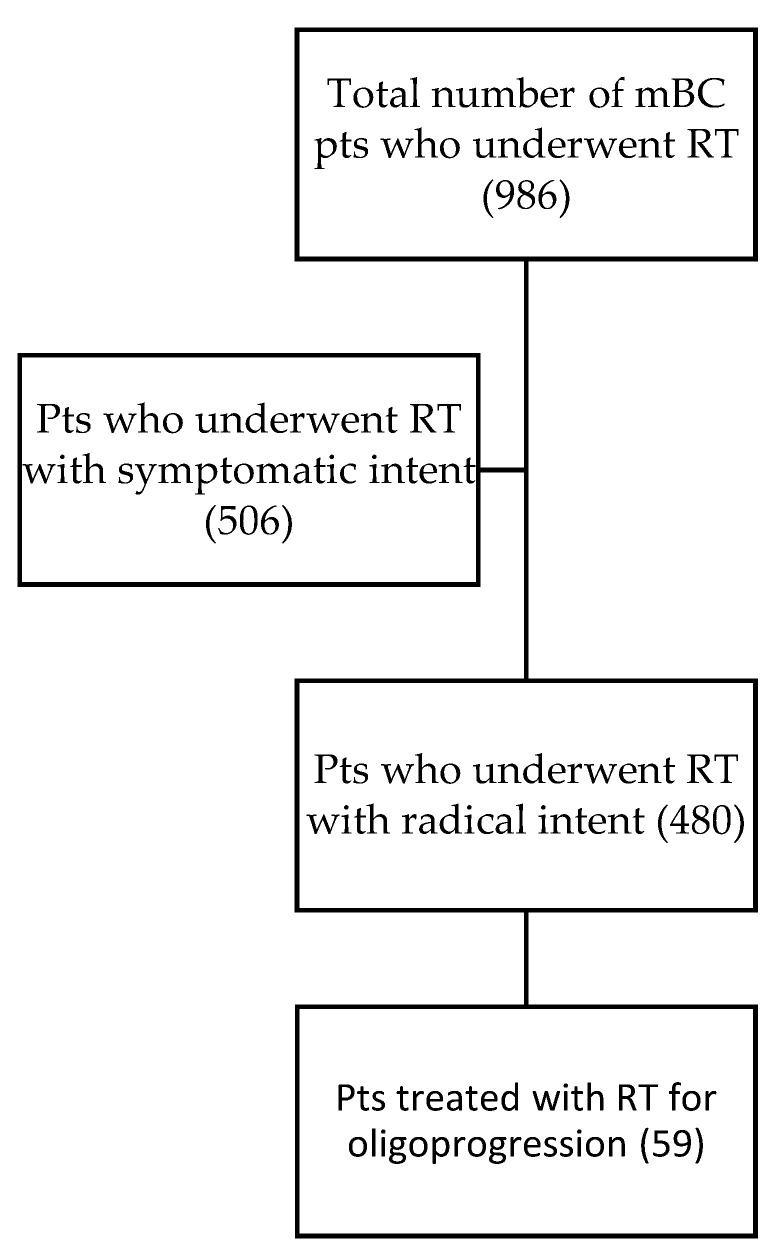
The CONSORT Diagram illustrates that among the 506 patients excluded for radiotherapy (RT) with symptomatic intent, all treatments were administered for palliative purposes in the context of plurimetastatic disease. Among the 480 patients treated with RT with a radical intent, those who underwent stereotactic body radiotherapy (SBRT) were treated for oligopersistent disease. In cases of disease progression, these patients also underwent a change in systemic therapy.

In our retrospective cohort study, we identified 59 patients using the GENERATOR Breast DataMart who met the inclusion criteria. These patients were considered oligoprogressive and received radiotherapy without altering their systemic therapy lines (Figure 1). Detailed characteristics of these patients can be found in Table 1.

### 3.2. Survival and Efficacy Analysis

**Figure 2:**
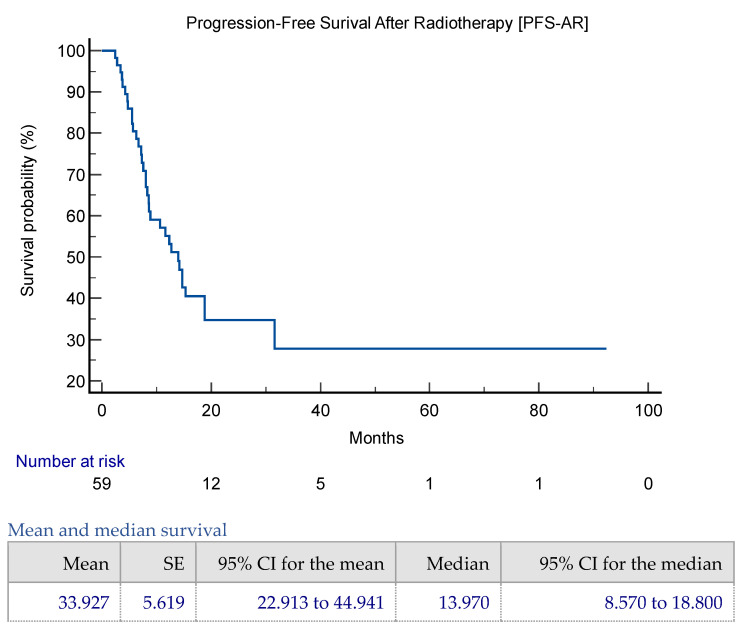

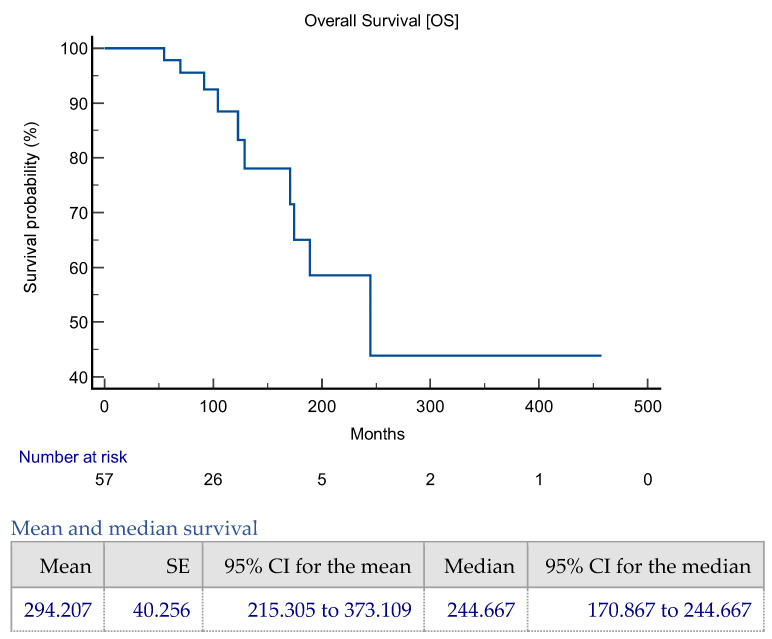
PFS-AR and OS Kaplan–Meier representation in the whole cohort.

**Figure A4:**
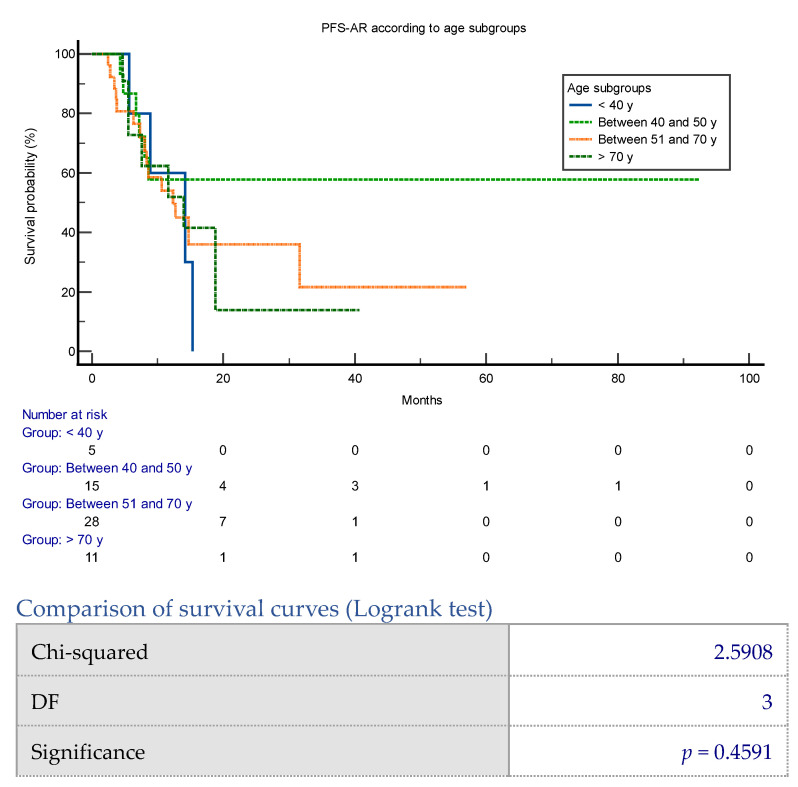
PFS-AR in patients with age < 40 years, between 40 and 50 years, between 51 and 70 years, >70 years.

The mean PFS-AR was 33.9 months (95% CI 22.9–44.9 months), while the median PFS-AR was 13.9 months (95% CI 8.5–18.8 months) (Figure 2). The rate of discontinuation of systemic therapy (RDT) was 3% (two patients) at three months and 18.6% (11 patients) at six months post-radiotherapy; these patients discontinued ongoing chemotherapy due to progressive disease. PFS-AR was not significantly associated with age (*p* = 0.45). Subgroup analysis showed median PFS-AR values of 14.1 months for patients under 40 years, not applicable (NA) for those between 40 and 50 years, 12.3 months for those between 51 and 70 years, and 13.9 months for those over 70 years (Figure A4).

**Figure A5:**
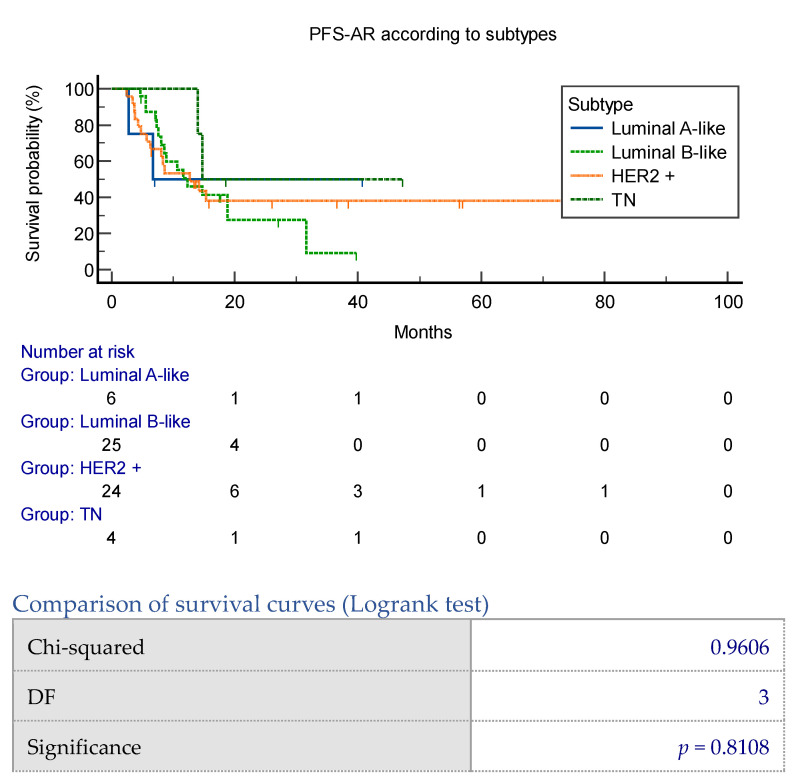
PFS-AR according to subtypes/subgroups.

**Figure A6:**
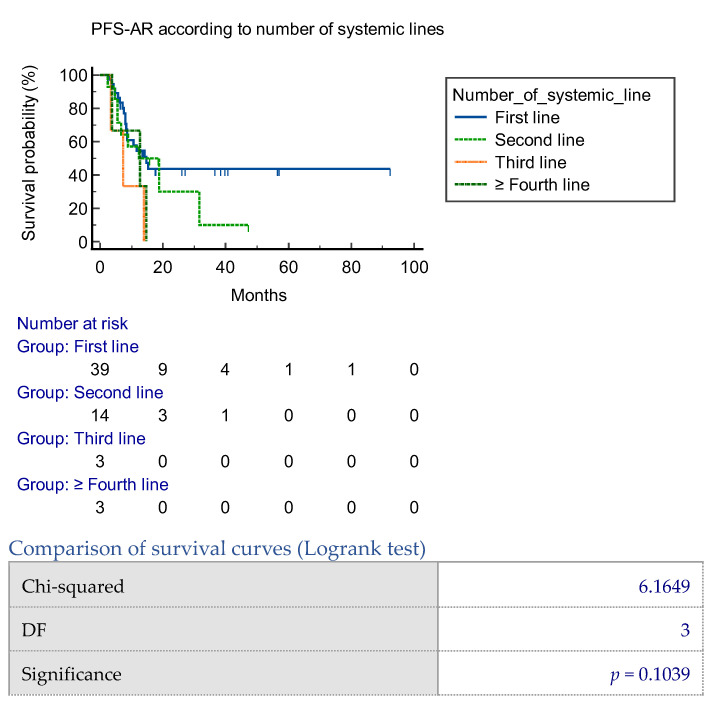
PFS-AR according to number of systemic lines ongoing.

Median PFS-AR varied across different breast cancer subtypes: 6.7 months for luminal A-like, 12.3 months for luminal B-like, 12.7 months for HER2+, and 14.7 months for triple-negative subtypes (*p* = 0.81) (Figure A5). When considering the number of ongoing systemic therapy lines, the median PFS-AR was 14.7 months for the first line, 12.3 months for the second line, 7.3 months for the third line, and 12.7 months for the fourth or more lines (*p* = 0.10) (Figure A6).

**Figure 3:**
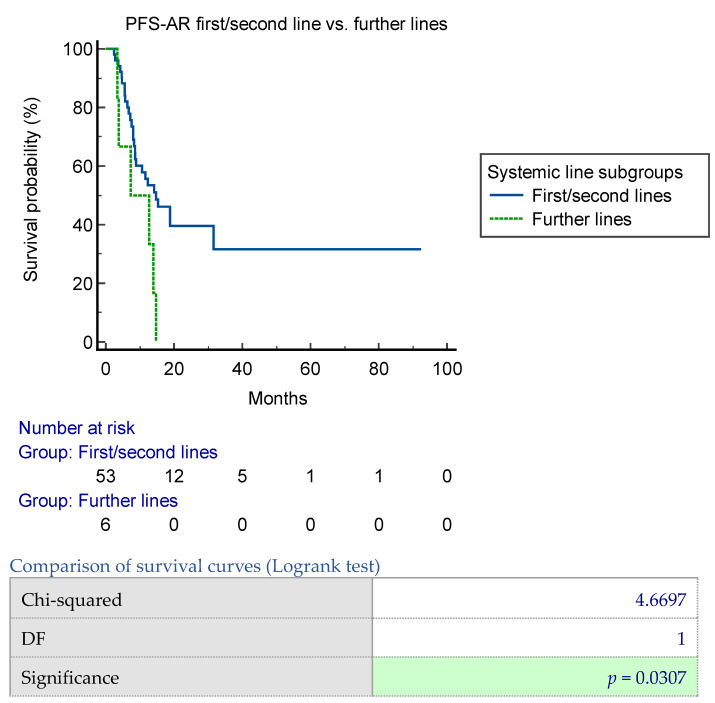
PFS-AR according to first/second line vs. further lines subgroups. PFS-AR was significantly better in first + second line vs. other lines analysis, with a median PFS 14.7 (95% CI 8.5–31.6), 7.3 (95% CI 3.4–14.7), respectively, for first + second lines and further lines groups [*p* = 0.03].

**Figure A7:**
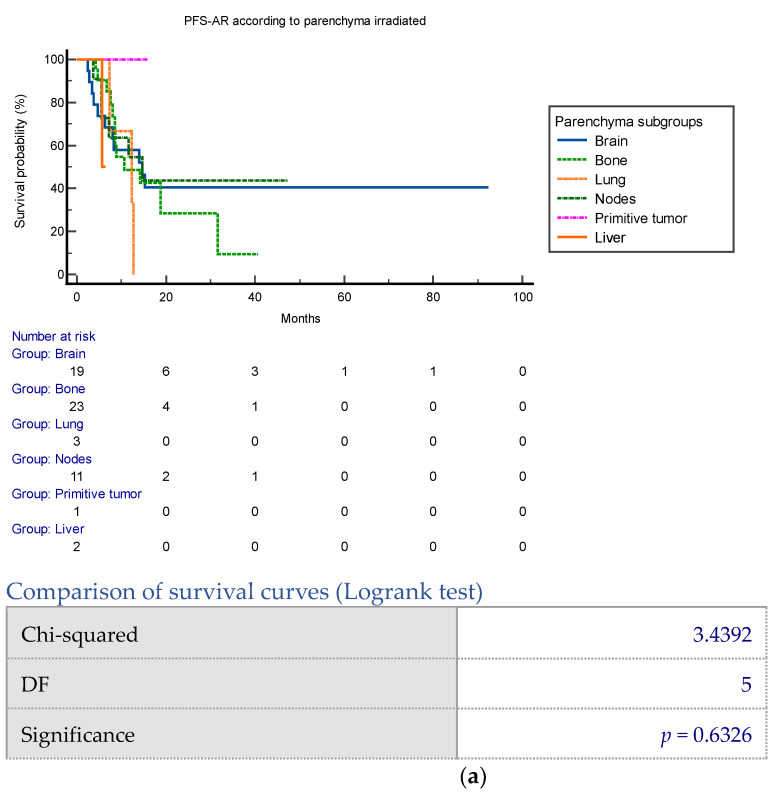

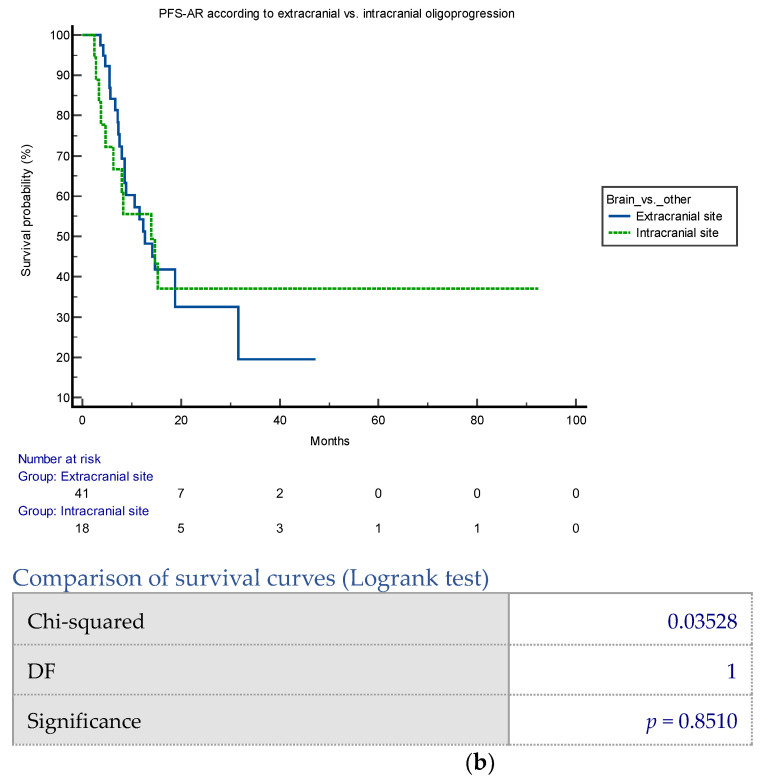
(**a**) PFS-AR according to parenchyma subgroups. (**b**) PFS-AR according to extracranial vs. intracranial subgroups.

A significant difference in the median PFS-AR was observed when comparing the first and second-line group with the subsequent lines group (*p* = 0.03) (Figure 3). Specifically, the median PFS was 14.7 months (95% CI 8.5–31.6) for the first and second-line group and 7.3 months (95% CI 3.4–14.7) for the subsequent lines group [*p* = 0.03]. Subgroup analysis on irradiated parenchyma did not show significance for all parenchyma and brain parenchyma vs. other sites (Figure A7a,b).

The median PFS-AR showed no significant difference between irradiated brain or other parenchyma. The median PFS was 14.7 months (95% CI 4.73–15.3) for the brain, 10.6 months (95% CI 8–31.6) for bone, 12.3 months (95% CI 7.3–12.7) for lung, 14.7 months (95% CI 5.53–14.7) for nodes, not reached (NR) for the primary tumor, and 5.67 months (95% CI 5.67–5.67) for liver (*p* = 0.63).

**Figure A8:**
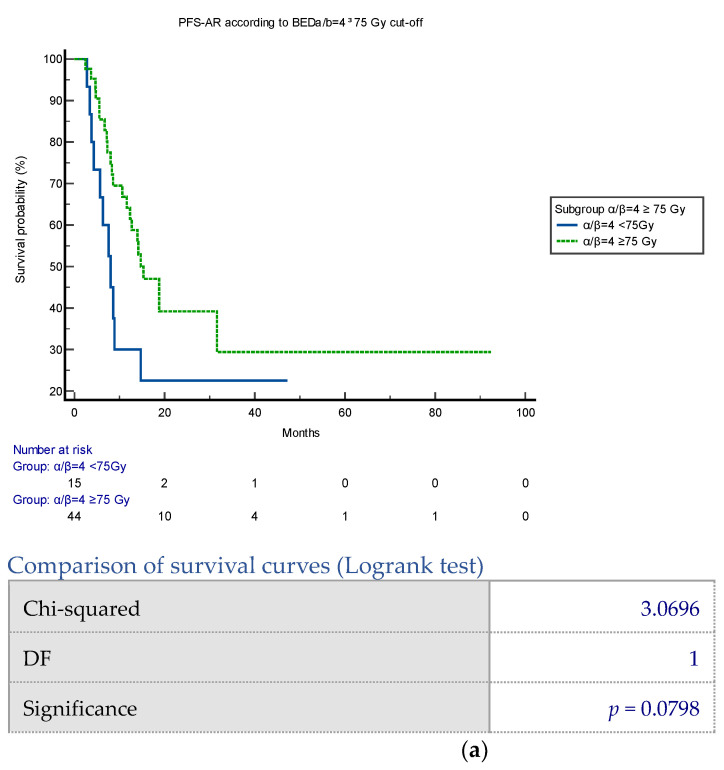

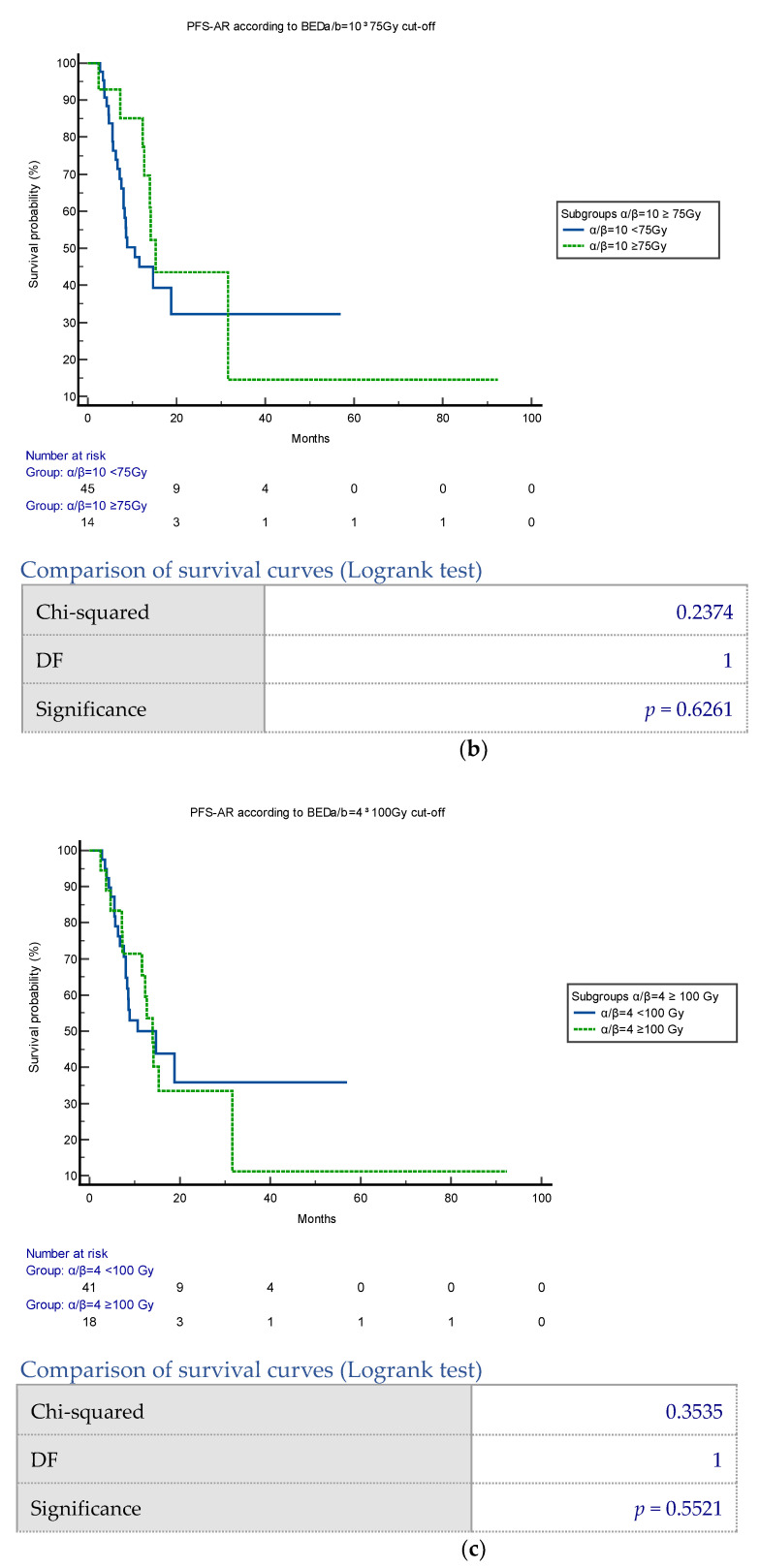

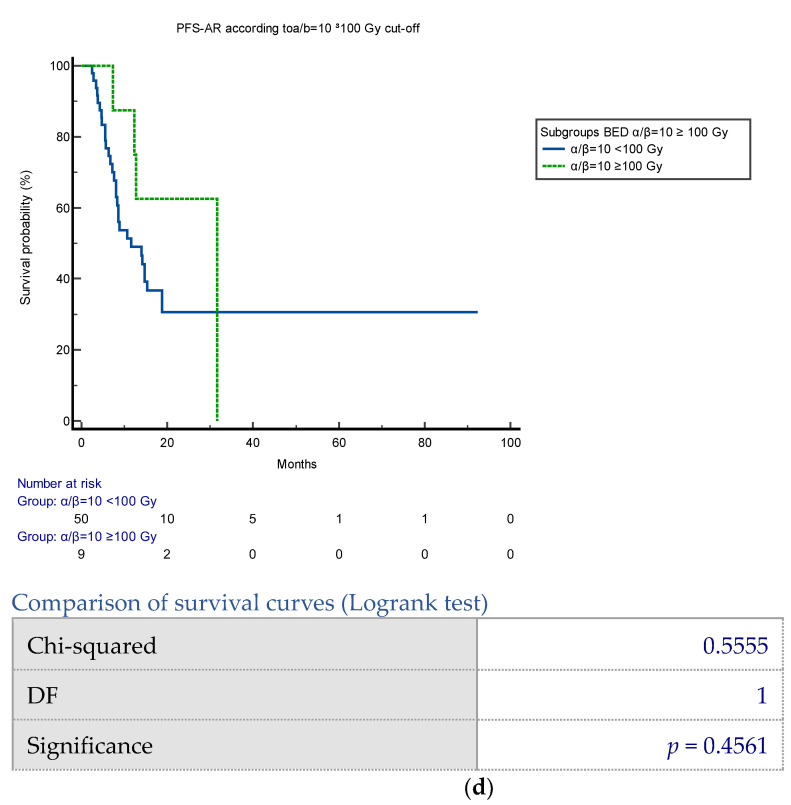
(**a**) Analysis of PFS-AR according to BED α/β = 4 ≥ 75 Gy cut-off. (**b**) Analysis of PFS-AR according to BED α/β = 10 ≥ 75 Gy cut-off. (**c**) Analysis of PFS-AR according to BED α/β = 4 ≥ 100 Gy cut-off. (**d**) Analysis of PFS-AR according to BED α/β = 10 ≥ 100 Gy cut-off.

PFS-AR was also not significantly different between brain and other irradiated parenchyma, with a median PFS of 12.7 months (95% CI 8.57–18.8) for other parenchyma and 13.9 months (95% CI 4.73–15.3) for brain (*p* = 0.85). Additionally, there were no significant correlations found for any BED subgroup regarding RT doses (Figure A8a–d).

PFS-AR did not show a significant difference between patients who received radiation doses of BED α/β = 4 ≥ 75 Gy, with a median PFS of 8 months (95% CI 3.7–14.7) for <75 Gy and 14.7 months (95% CI 10.6–31.6) for ≥75 Gy (*p* = 0.07).

The difference in progression-free survival (PFS-AR) was not statistically significant for patients who received radiation doses of BED α/β = 10 ≥ 75 Gy. The median PFS was 10.6 months (95% CI 7.57–18.8) for <75 Gy and 15.3 months (95% CI 12.3–31.6) for ≥75 Gy (*p* = 0.62). Similarly, for BED α/β = 4 ≥ 100 Gy, the median PFS was 14.7 months (95% CI 8–18.8) for <100 Gy and 13.9 months (95% CI 7.3–31.6) for ≥100 Gy (*p* = 0.55). When the BED α/β = 10 ≥ 100 Gy, the median PFS was 11.6 months (95% CI 8–18.8) for <100 Gy and 31.6 months (95% CI 7.3–31.6) for ≥100 Gy (*p* = 0.45).

The study found that in the multivariate COX regression analysis for PFS-AR, BED with α/β = 4 > 100 Gy was the only significant variable (*p* = 0.0017, C-index 0.687, 95% CI 0.59–0.78) (Table 2). The median overall survival (OS) was 244 months (95% CI 170–244 months) (Figure 2). No patients died or discontinued chemotherapy due to radiotherapy toxicity. Additionally, an abscopal effect was observed in one patient with the HER2+/ER− subtype who underwent lung SBRT. This patient experienced a complete response not only in the irradiated lung metastases but also in pleural disease during Pertuzumab and Trastuzumab therapy. Further details are provided in Appendix A.

## 4. Discussion

Oligoprogression is a relatively new concept in clinical practice. Clinicians are encountering it more often, especially with the development of new targeted treatments and advanced imaging technologies [13]. Currently, there is significant scientific interest in this area, aiming to improve survival outcomes by combining local treatments with systemic therapies. While some studies have shown promising results, especially in lung cancer with EGFR mutations, there are fewer available data for prostate cancer and renal cell carcinoma [20,21,22]—Table 3

The use of radiotherapy to treat oligoprogression in metastatic breast cancer (mBC) has been relatively underexplored in the literature [1,6,23,24]. Kam et al. conducted a retrospective study involving a mixed cohort of patients with oligometastatic (12 patients) and oligoprogressive (10 patients) cases who were treated with stereotactic body radiotherapy (SBRT) for skeletal metastases. It was found that breast cancer accounted for 32% of the cases, and the reported oligoprogressive progression-free survival (PFS) was 6.6 months over a median follow-up of 15.6 months. Additionally, the results of the phase II randomized CURB trial, which investigated SBRT administration in a mixed cohort of lung (59 patients) and breast cancer patients (47) with oligoprogression, were presented at ASCO 2022.

The trial showed that patients who received both radiotherapy on oligoprogressive sites and systemic therapy had a significantly longer progression-free survival of 31 weeks (7 months) compared to those who only received chemotherapy. The mean follow-up period for the study was 44 weeks. Our study, which involved 59 patients with oligoprogressive metastatic breast cancer, is the only one based on real-world data. It included a comprehensive literature review and demonstrated an impressive median follow-up of over three years.

In our study, we found promising results regarding the potential benefits of radiotherapy for treating oligoprogressive metastatic breast cancer. The average progression-free survival (PFS) after radiotherapy was 13.7 months (95% CI 8.6–18.8 months), with an average follow-up duration of 38 months. Notably, only 18.6% (11 patients) required a change in systemic treatment due to disease progression after six months. These findings indicate that radiotherapy may have a significant role in prolonging progression-free survival and enhancing disease control in this patient population.

In clinical practice, the use of radiotherapy (RT) to treat oligoprogression is a well-established guideline indication for certain selected patient populations, such as those with oncogene-addicted lung cancer [12]. However, the role of RT in the context of breast cancer remains under investigation. This is likely due to the limited evidence available and a lack of clear guidelines regarding which patient subsets may benefit from this approach. With advancements in biomolecular and targeted therapies, like CDK4/6 inhibitors for metastatic luminal-like disease and the combination of Pertuzumab and Trastuzumab for metastatic HER2+ disease [25,26,27,28,29,30], there is a growing potential to enhance progression-free survival (PFS) by improving disease control. Our analysis of subgroups did not show a significant association between progression-free survival after radiotherapy (PFS-AR) and breast cancer subtypes. The median PFS values were 6.7 months (95% CI 2.7–6.7) for luminal A-like, 12.3 months (95% CI 8.0–18.8) for luminal B-like, 12.7 months (95% CI 6.3–15.3) for HER2+, and 14.7 months (95% CI 13.9–14.7) for triple-negative subtypes, with a *p*-value of 0.81.

In our study, we observed that one patient with triple-negative breast cancer had a poor prognosis with rapid disease progression at the three-month follow-up. On the other hand, another patient with triple-negative breast cancer, who has a BRCA mutation, is still alive with no sign of disease. This suggests that radiotherapy, taking into account the patient’s BRCA mutation status, may be beneficial. Our findings indicate that HER2+ breast cancer and some cases of luminal-like metastatic breast cancer may benefit from stereotactic body radiotherapy (SBRT) for oligoprogression. However, triple-negative breast cancer, which was not well-represented in our study and demonstrated rapid disease progression, may not be as suitable for ongoing systemic therapy due to its aggressive nature.

In a recent review by Patel et al. [13], mechanisms of resistance in HER2+ oligoprogressive disease are hypothesized, and a potential role for RT in this setting is postulated. Our data, which could potentially change clinical indication to local therapies in oligoprogressive breast cancer, support the notion that HER2+ subtype breast cancer patients could indeed benefit from SBRT in the oligoprogressive setting, particularly during first-line therapy.

In our study, we found that the type of ongoing systemic therapy was significantly related to progression-free survival after radiotherapy (PFS-AR). Particularly, patients treated for oligoprogression during their first and second systemic therapy had longer PFS-AR. Their median PFS values were 14.7 months (95% CI 8.5–31.6) and 7.3 months (95% CI 3.4–14.7) for the first + second lines and further lines groups, respectively (*p* = 0.03). This finding may be due to the heterogeneity of metastatic breast cancer. Research has shown that resistant metastatic deposits undergo clonal expansion and diversification, acquiring additional driver alterations before becoming clinically detectable. Early detection of therapeutic resistance is crucial for optimizing therapy. Detecting isolated sites of resistance to systemic therapies early allows for their continuation, especially when combined with a local treatment aimed at eradication.

Concerning the radiotherapy (RT) treatments in our study, there was variability in volumes and doses due to the retrospective nature of the analysis. Standardizing doses by converting them to biologically effective doses (BEDs) at α/β = 4 and α/β = 10 did not reveal a clear advantage for specific dose levels in treating oligoprogressive sites, with the exception of a positive trend towards improved PFS-AR in patients treated with BED > 75 Gy at α/β = 4 (*p* = 0.07). Furthermore, multivariate analysis identified a BED at α/β = 4 > 100 Gy as the sole significant factor related to PFS-AR. This suggests that gaps still exist in our understanding of the radiobiological behavior of oligoprogressive disease, including aspects such as immunomodulation and potential synergies with ongoing targeted therapies. From the existing literature, a recent review on breast cancer bone metastases highlighted a favorable threshold dose of BED > 75 Gy in oligometastatic cancer, associated with improved overall survival. These findings indicate the need for further research to clarify the optimal radiation doses and their impact on outcomes in the context of oligoprogressive disease.

Furthermore, brain metastases were well represented, accounting for 55.5% of cases. However, an analysis based on the site irradiated (brain vs. non-brain) did not reveal a difference in progression-free survival after radiotherapy (PFS-AR). This suggests that the efficacy of radiotherapy in oligoprogressive disease may be more closely linked to disease control at sites of resistance, regardless of the type of parenchyma involved. It is important to acknowledge the limitations of this study, including its retrospective nature, which led to heterogeneous data collection, particularly in terms of patient enrollment, subtype distribution, parenchyma irradiated, and radiotherapy dose and volumes. Additionally, the cohort size was limited to only 59 patients selected from our cases over the last 8 years. Despite these limitations, the data indicate that radiotherapy administration for oligoprogressive breast cancer may confer an advantage in terms of PFS-AR, especially in patients receiving treatment during the first systemic line in HER2+ and luminal-like disease subtypes.

**Table 3:** Summary of literature review of oligoprogressive breast cancer and radiotherapy.

Author	N° PTS PTS Characteristics	Dose and Volumes	PFS	Local Control	OS
Kam TY, 2019 [30]	22 pts Different solid tumors (MBC 7 pts tot) Both oligometastatic and oligoprogressive	Both spinal and non-spinal metastases between 35 and 50 Gy, respectively, in five fractions	6.6 months in OP group	1-year LC 91.2%	Not reported
Shahi J, 2020 [31]	52 pts Different solid tumors (MBC 11 pts tot) Both local progressions, oligometastatic and oligoprogressive	SBRT dose was 35 Gy (range, 30–50 Gy in 5 fr) with a median biologically effective dose of 59.5 Gy (range, 48–100 Gy)	Median PFS was 4.0 months (95% confidence interval, 2.8–7.3)	LF was 9.0% at 2 years	OS was 31.7 months (95% confidence interval, 23.8–87.5)
Tsai TJ, 2021 CURB Trial [32]	102 pts Different solid tumor (44 MBC, 22 for each arm of randomization) Patients were randomized 1:1 between SBRT to all progressive sites plus palliative standard of care (SOC) vs. palliative SOC only	NA	No difference in median PFS was seen in the breast cohort (18 weeks with SBRT vs. 17 weeks with SOC; *p* = 0.5).	NA	NA
Ramadan S, 2022 [33]	81 pts Different solid tumor (12 MBC tot) Only olioprogressive pts	SBRT dose (Gy) 40 (18–60) SBRT in five fractions (2–8)	Median PFS was 7.8 (95% CI 4.6–10.9) months	Local failurewas 5% at 1 year and 7.3% at 2 years	Median OS was 25.1 (95% CI 11.2–39.1) months
Weikamp F, 2020 [34]	46 pts Only MBC Both oligometastatic and oligoprogressive	Median biologically effective dose (BED at α/β = 10) was 81.6 Gy (range: 45–112.5 Gy)	Median PFS at 2 years was 17%	At 2 years, local control (LC) was 89%	Median OS at 2 years was 62%
Nicosia L, 2022 [35]	79 pts Only MBC Only oligoprogressive	Only SBRT	NA	The 2-year FLP in the overall population was 86.7%	NA
Tan H, 2021 [36]	129 pts Only MBC Both oligometastases, oligoprogression, and local control of dominant tumor (CDT)	Extra-cranial SBRT to metastatic lesions	1-year PFS for oligoprogression, was 19.6%	1- and 2-year LC rates were 89% and 86.6%	1- and 2-year OS rates were 83.5% and 70%

## 5. Conclusions

This retrospective study explores the potential benefits of radiotherapy (RT) in treating oligoprogressive metastatic breast cancer (mBC). It suggests that RT, particularly when administered alongside first-line systemic therapies, may lead to improved survival, especially in patients with HER2+ and luminal-like disease subtypes. The study is significant because it represents the only reported group with oligoprogressive breast cancer treated with radiotherapy and possessing such a long follow-up duration. The results from this exploratory study should be confirmed in an independent larger patient cohort which may subsequently pave the way for prospective trials to confirm the role of RT in oligoprogressive mBC. These trials will aim to identify the optimal setting, timing for combining systemic therapies, and the appropriate dose of RT treatments. Since patients receiving ongoing first- and second-line systemic therapies have shown an advantage in progression-free survival (PFS), it is recommended to design a randomized phase II trial with a control arm targeting this specific patient population. The main limitation of the study is represented by retrospective analysis and heterogeneous cohort analysis. It is important to note that a control arm was not available for this retrospective analysis, as its primary intent was to describe the cohort of patients who underwent radiotherapy without changing systemic drugs. Moving forward, well-designed prospective trials will be essential for further elucidating the efficacy and potential benefits of RT in oligoprogressive mBC.

## Figures and Tables

**Table 1:** Patients’ and treatments’ characteristics.

Epidemiologic Characteristics		
Age	Median 57 (34–86)	
Age subgroups	<40 years	5 pts (8%)
	Between 40–50	15 pts (25%)
	Between 51–70	28 pts (48%)
	>70 years	11 pts (19%)
Metastatic disease esordium	At diagnosis de novo	13 pts (22%)
	Distant relapse	46 pts (78%)
Subtype	Luminal A-like	6 pts (10.2%)
	Luminal B-like	25 pts (42.3%)
	HER2+	24 pts (40.7%)
	Triple negative	4 pts (6.8%)
HER2+ population	HER2+/ER+	19 pts (76%)
	HER2+/ER−	6 pts (24%)
Parenchymal site of oligoprogression	Brain	19 pts (32.2%)
	Bone	23 pts (39%)
	Lung	3 pts (5.1%)
	Nodes	11 pts (18.6%)
	Primitive tumor	1 pt (1.7%)
	Liver	2 pts (3.4%)
Systemic Therapies Characteristics		
Systemic therapies ongoing	Anti-HER2	24 pts (40.7%)
	CDK4/6i	17 pts (28.8%)
	Endocrine therapies	12 pts (20.4%)
	Chemotherapy	6 pts (10.1%)
Anti-HER2 therapies	Pertuzumab + Trastuzumab	13 pts (54.1%)
	T-DM1	6 pts (25%)
	Trastuzumab + Capacitabine	2 pts (8.3%)
	Lapatinib + Capecitabine	1 pt (4.2%)
	Trastuzumab + Vinorlbine	1 pt (4.2%)
	Trastuzumab + Hormone therapy	1 pt (4.2%)
Number of systemic line ongoing	First	39 pts (66.1%)
	Second	14 pts (23.7%)
	Third	3 pts (5.1%)
	≥Fourth	3 pts (5.1%)
Radiotherapy Characteristics		
Radiotherapy techniques	3D-CRT	9 pts (15.6%)
	IMRT	1 pt (1.7%)
	VMAT/SBRT	49 pts (83%)
Bone RT treatments	SIB 40–30/20 Gy in 5 fr	16 pts (69.5%)
	3D 30 Gy in 10 fr	3 pts (13%)
	3D 20 Gy in 5 fr	3 pts (13%)
	Re-RT 16 Gy in 14 fr BID	1 pts (4.5%)
Brain RT treatments	SBRT 25.5 Gy in 3 fr	10 pts (52.6%%)
	WB-SIB 50/30 Gy in 10 fr	7 pts (36.8%%)
	3D WB 30 Gy in 10 fr	1 pt (5.3%)
	Re-RT WB 16 Gy in 14 fr	1 pt (5.3%)
Lung RT treatments	SBRT 50 Gy in 5 fr	3 pts (100%)
Nodes RT treatments	SBRT 50–35 Gy in 5 fr	8 pts (72.7%)
	SIB 57.5/50 Gy in 25 fr	1 pt (9.1%)
	Re-RT 38 Gy in 30 fr	1 pt (9.1%)
	3D 28 Gy in 16 fr	1 pt (9.1%)
Liver RT treatments	SBRT 40 Gy in 5 fr	1 pt (100%)
Primitive tumor RT treatments	SBRT 24 Gy in 3 fr	1 pt (100%)
BED schedule analysis(For every analysis, patients were grouped according to dichotomous categories, resulting in more than one category)	RT with schedules ≥100 Gy for α/β = 10	9 pts
	RT with schedules ≥100 Gy for α/β = 4	18 pts
	RT with schedules ≥75 Gy for α/β = 10	14 pts
	RT with schedules ≥75 Gy for α/β = 4	44 pts

**Table 2:** Cox proportional regression analysis for multivariate analysis of PFS-AR prediction.

Covariate	*p*	Exp(b)	95% CI of Exp(b)
Age subgroups	0.9772	0.9929	0.6096 to 1.6173
First + second line vs. further lines	0.9570	1.0700	0.0918 to 12.4737
Subtype	0.1797	0.6850	0.3941 to 1.1905
Number of systemic lines	0.3384	1.5573	0.6288 to 3.8569
Parenchyma irradiated	0.3263	0.7809	0.4765 to 1.2796
BED 100 a/b = 10	0.5289	0.5931	0.1167 to 3.0153
BED 75 a/b = 10	0.0858	0.1720	0.0231 to 1.2812
BED 100 a/b = 4	0.0017	9.9406	2.3737 to 41.6289
BED 75 a/b = 4	0.0984	0.4053	0.1389 to 1.1829
Brain vs. other parenchyma	0.8739	1.1106	0.3038 to 4.0604
Harrell’s C-index		0.687	
95% Confidence interval		0.593 to 0.781	

**Figure A1:**
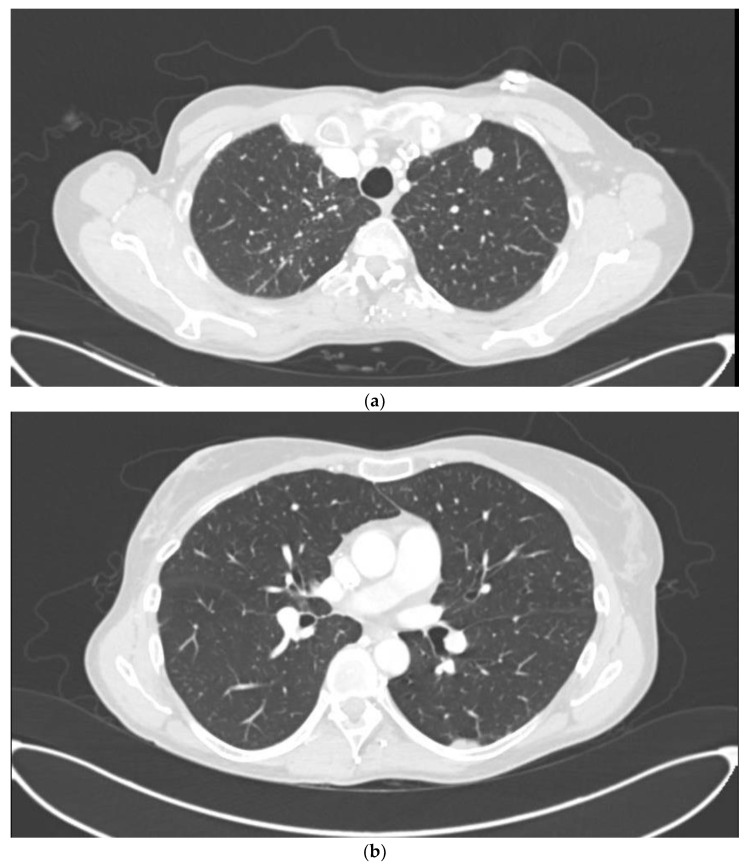
CT basal on September 2015: lung metastasis in in left superior lobe (LSL) (**a**) and an ipsilateral pleural plaque in the superior segment of inferior lobe (**b**).

**Figure A2:**
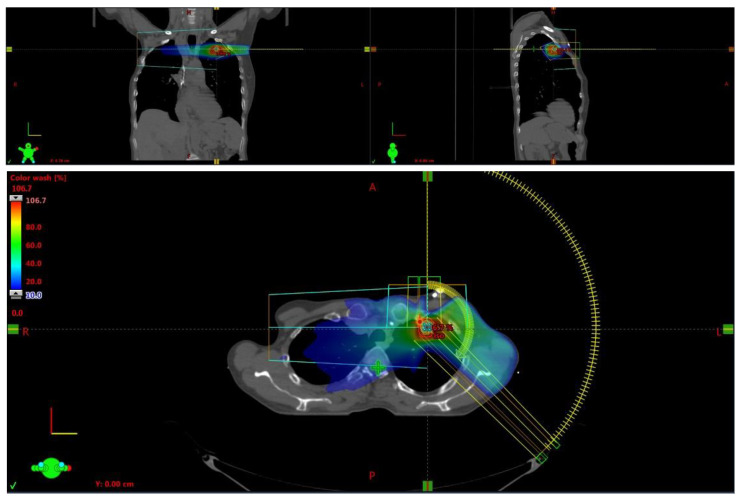
Images of treatment plan for lung metastases.

**Figure A3:**
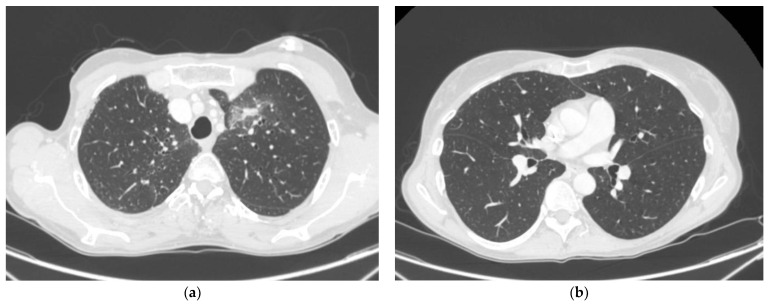
CT of re-evaluation on February 2016 with RT fibrosis at site treated with SBRT (**a**) and complete distant response on pleural plaque (**b**).

## Data Availability

All data generated and analyzed during this study are included in this published article.

## Appendix A

In this retrospective series, an intriguing case of an abscopal effect following radiotherapy was observed in a 52-year-old patient diagnosed with stage IIIA breast cancer in 2011 (ER 0%, PR 0%, Ki67 80%, HER2+). Following diagnosis, the patient underwent adjuvant chemotherapy with TCH schedules. Subsequently, in 2012, metastases were detected in the lungs and thoracic nodes, prompting initiation of systemic therapy with Trastuzumab. In March 2015, disease progression was noted on instrumental exams, revealing oligoprogression in a lesion in the left superior lobe (LSL), alongside an ipsilateral pleural plaque in the superior segment of the inferior lobe. Given the overall disease status, the patient underwent stereotactic body radiotherapy (SBRT) for the lung metastases, receiving a dose of 50 Gy in five fractions. Radiotherapy was administered in September 2015, while the patient continued receiving TDM1 as systemic therapy. Remarkably, upon re-evaluation in February 2016, a complete response was observed not only in the irradiated lung lesion but also in the pleural plaque, with stable disease in the thoracic nodes. The patient continued TDM1 until October 2016 when progression was detected in the contralateral lung, resulting in a progression-free survival after radiotherapy of 17 months. This case highlights the potential of radiotherapy to induce an abscopal effect, leading to regression of distant untreated lesions, and underscores the importance of further exploring this phenomenon in the context of metastatic breast cancer treatment.

## Appendix B

We report additional subgroup analysis.
